# The Opening Session

**Published:** 1898-10-08

**Authors:** 


### The Opening Session.

With the coming of another October we find ourselves once more at that most interesting point in the medical year, when all the workers reassemble after the holidays and again take up the thread of their daily life. The commencement of the winter session means something more than the mere reopening of the schools. It means the reassembling of the societies, the reporting of cases, the discussion of results, the subjection of discoveries to criticism, and all that friction of mind against mind which makes for progress. Quite apart, then, from the human interest that must always attach to a season at which a large number of young people leave behind them their boyhood and take their first step in the career which is to give them their position among men, there is much in the circumstances of October to mark it as a period of reawakening in the medical world. In his introductory address at St. George's Hospital, Mr. G. R. Turner did well to point out how vastly different a being the medical student of to-day is from the vulgar person so amusingly and graphically described by Dickens and by Thackeray as the type of the so-called medical student of a bygone age. The persistence with which people of every condition in life have held to that conception of the medical student which portrays him as uneducated and unclean, and as characterised by a low fastness redeemed only by the saving grace of humour and a love of practical jokes, while, no doubt, a striking compliment to the power of certain writers of the first half of the century, is for all that a distinct hardship to the medical student of to-day, who, even now, after all these years, has still to live down the ill-name given him so long ago. It must be remembered, however, that at the present time, and we may hope it will be still more the case in the future, the students are largely recruited from among public school and university men. Mr. Turner says that at Cambridge, out of the 9,437 men who have matriculated during the past ten years, no fewer than 1,253, *i.e.*, nearly one in every seven, were registered students of medicine.

We are perhaps apt to forget, and certainly many people in discussing the University of London question did forget, that, whether we will or no, London is already a great university town, having about 3,000 students attending at the various colleges which have to do with the one subject of medicine alone, and, as Mr. Turner says, the behaviour of these students "compares not unfavourably with the admittedly well-behaved undergraduates of Oxford and Cambridge, who have less temptation and more restraint."

The fascination of such a scientific study ought indeed to attract the most intelligent of the well-to-do, while the extent to which medicine is allied with philanthropic work ought to claim for it the highest State and social recognition.

The philanthropic side of the medical profession is, indeed, one to which it is necessary to call attention, even though, in the language of the copy-books, self praise be no commendation. "The public by their liberality," says Mr. Turner, "have hitherto prevented the institution of State-aided hospitals, and now support by voluntary contributions 716 hospitals in the British Isles. Dispensaries and convalescent homes swell the number to 1,155 institutions, spending, according to Sir H. Burdett, nearly £3,000,000 a year." All this, surely, shall be counted to them for righteousness. But if so, shall not the enormous amount of help given by the medical profession in the conduct of these charities be counted to them also? Let us look at the figures. At the various general hospitals in London, with an expenditure of nearly half a million pounds a-year among them, the total payment distributed among the 165 resident and 466 non-resident medical men by whom the work is done amounts to a sum of less than £13,000. That is to say, of the sum expended by these charities, the very essence of whose work is the giving of medical aid, only a little over 2½ per cent. is paid for the medical aid so given. Only four hospitals pay anything to their visiting staff, while the other eight hospitals pay—and pay inadequately—only 38 out of their 240 medical officers.

The probability is that facts like these are unknown to the majority of those outside the profession, nor is the enormous amount of private charity in the form of gratuitous advice, which is given by general practitioners, at all appreciated as it should be, but taking it altogether there can be no doubt that, if only for the gifts in kind which are made by medical men every day, the medical profession should stand high among the philanthropic institutions of the country.

